# Screening for Potential Novel Probiotics With Dipeptidyl Peptidase IV-Inhibiting Activity for Type 2 Diabetes Attenuation *in vitro* and *in vivo*

**DOI:** 10.3389/fmicb.2019.02855

**Published:** 2020-01-10

**Authors:** Fenfen Yan, Na Li, Yingxue Yue, Chengfeng Wang, Li Zhao, Smith Etareri Evivie, Bailiang Li, Guicheng Huo

**Affiliations:** ^1^Key Laboratory of Dairy Science, Ministry of Education, Northeast Agricultural University, Harbin, China; ^2^Food College, Northeast Agricultural University, Harbin, China; ^3^Food Science and Human Nutrition Unit, Department of Animal Science, University of Benin, Benin City, Nigeria

**Keywords:** *Lactobacillus acidophilus*, DPP-IV, antioxidative, diabetes, probiotic, antidiabetic

## Abstract

Diabetes has become the second most severe disease to human health. Probiotics are important for maintaining gastrointestinal homeostasis and energy balance and have been demonstrated to play a positive role in the prevention and treatment of metabolic syndromes, such as obesity, inflammation, dyslipidemia, and hyperglycemia. The objective of this study was to screen potential antidiabetic strains *in vitro* and evaluate its effects *in vivo*. For the *in vitro* section, dipeptidyl peptidase IV (DPP-IV) inhibitory and antioxidant activities of 14 candidate *Lactobacillus* spp. strains were tested. Then hydrophobicity and acid and bile salt tolerance assays were determined. The most promising *in vitro* strain was further evaluated for its antidiabetic properties *in vivo* using type 2 diabetes mice induced by high-fat diet and intraperitoneal injection of streptozotocin (STZ). The reference strain for this study was *Lactobacillus rhamnosus* GG. Results showed that cell-free excretory supernatants and cell-free extracts of *Lactobacillus acidophilus* KLDS1.0901 had better DPP-IV inhibitory activity, antioxidative activities, and biological characteristics than other strains. At the end of the treatment, we found that *L. acidophilus* KLDS1.0901 administration decreased the levels of fasting blood glucose (FBG), glycosylated hemoglobin, insulin in serum and AUC_glucose_, and increased the level of glucagon-like peptide 1 in serum compared with diabetic mice (*p* < 0.05). Moreover, *L. acidophilus* KLDS1.0901 supplementation increased the activities of superoxide dismutase, glutathione peroxidase, the level of glutathione, and reduced the level of malondialdehyde in serum. These results indicated that *L. acidophilus* KLDS1.0901 could be used as a potential antidiabetic strain; its application as food supplement and drug ingredient is thus recommended.

## Introduction

Diabetes remains a global health problem today, with sufferers having either type 1 diabetes (T1D, insulin-dependent) or type 2 diabetes (T2D, non-insulin-dependent) (Imamura et al., 2011). According to the International Diabetes Federation (IDF), the global prevalence of diabetes in adults is about 8%, which is projected to increase to >10% by 2040 (Lascar et al., 2018). Diabetes sufferers face high risks of many other life-threatening challenges, often leading to high medical costs, lowered life qualities, and early mortality statistics (Cho et al., 2018). T2D is a multifactorial disorder caused by genetic, epigenetic, and environmental factors. The deficiency in islet β-cell secretion, insulin resistance, hyperglycemia, systemic inflammation, and oxidative stress is the important pathophysiological manifestations (Mengual et al., 2010; Donath and Shoelson, 2011; Cani, 2012).

A number of antidiabetic agents have been developed over the years to treat T2D, with dipeptidyl peptidase IV (DPP-IV) inhibitors being one of the most recent agents to combat it (Gherardo et al., 2011; Akoumianakis et al., 2018; Musoev et al., 2019). They function by enhancing the incretins effect of glucose-dependent insulinotropic polypeptide (GIP) and glucagon-like peptide-1 (GLP-1) (Karagiannis et al., 2012). These incretins could promote glucose-dependent insulin secretion and suppress the release of pancreatic glucagon and slow gastric emptying (Thoma et al., 2003; Verdenelli et al., 2009). To enhance the effect of GLP-1, a number of synthetic DPP-IV inhibitors (vildagliptin, saxagliptin, alogliptin, and linagliptin) have been developed, having fewer side effects compared to many traditional antidiabetic agents (Bo et al., 2000; Reimer et al., 2002). DPP-IV inhibitors from natural sources seem to be more safe and desirable. Interestingly, some recent studies have indicated that some *Lactobacillus* spp. strains could inhibit DPP-IV activity (Zhu et al., 2016).

A large number of studies have reported that the oxidative stress status of the body was upregulated in diabetic patients than that in normal subjects (Jain, 1989; Bloch-Damti and Bashan, 2005; Wang X. et al., 2017). Hyperglycemia, a typical clinical characteristics of diabetes, could increase the levels of oxidative stress markers, which was positively related to blood glucose and HbA1c levels in diabetic patients (Jain et al., 1989; Kolati et al., 2015; Behl et al., 2016). Additionally, previous studies have also reported that oxidative stress could cause insulin resistance and impair β-cell structure and function and result in T2D, but the molecular mechanisms are still unclear (Eriksson, 2007). One of the important impaired mechanisms was inducing insulin receptor substrate (IRS) serine/threonine phosphorylation, disturbing insulin signaling by reactive oxygen species (ROS) (Morino et al., 2006). Previous studies have revealed that a number of strains of *Lactobacillus* had antioxidative activity *in vitro* (Chen et al., 2014c; Tang et al., 2017) and significantly decreased the oxidative stress *in vivo*, showing antidiabetic effects (Ejtahed et al., 2012; Singh et al., 2016; Kumar et al., 2017). Based on current research states, the application of probiotics to ameliorate T2D has bright prospects.

In recent years, probiotics have been used in developing functional foods or dietary supplements (Kerry et al., 2018; Betoret et al., 2019). Probiotics are defined as “live microorganisms which when administered in adequate amounts confer one or more health benefit on the host” (Araya et al., 2015). Recent data suggest that the administration of probiotics, especially *Lactobacillus* substrains, can prevent or delay the onset of diabetes by decreasing the level of blood glucose, HbA1c, insulin resistance, and oxidative stress in animal experiments and clinical trials (Zhang et al., 2016; Kim et al., 2017; Liu et al., 2017; Tonucci et al., 2017b). *Lactobacillus rhamnosus* GG is an important commercial strain with good biological characteristics (acid and bile salt tolerance and cell adhesion) and probiotic properties (antioxidant and anti-inflammatory activity). Additionally, a study has demonstrated that *L. rhamnosus* GG could reduce blood glucose and HbA1c levels and increase insulin sensitivity in streptozotocin (STZ)-induced diabetic rats compared with diabetic rats (Tabuchi et al., 2003; Groele et al., 2017). Assessing potential novel strains that possess the abovementioned features is thus necessary to broaden the use of probiotics in treating T2D.

The present study thus aims to screen potential antidiabetic *Lactobacillus* spp. strains based on DPP-IV inhibitory activity, antioxidative activity, and biological characteristic including acid and bile salt tolerance and cell surface hydrophobicity *in vitro* and evaluate antidiabetic effects in T2D mice induced by high-fat diet (HFD) and intraperitoneal injection of STZ.

## Materials and Methods

### Chemicals and Reagents

Gly-Pro-*p*-nitroanilide and DPP-IV were purchased from Sigma Chemical (St. Louis, MO, United States). Diprotin A was obtained from Shanghai Qiangyao Bioengineering Institute (Shanghai, China). All other chemicals and reagents were purchased from the Tianli Chemical Reagent Company (Tianjin, China) unless otherwise stated. Kits used to measure the levels of superoxide dismutase (SOD), GSH peroxidase (GSH-Px), glutathione (GSH), and malondialdehyde (MDA) were purchased from the Nanjing Jiancheng Bioengineering Institute (Nanjing, China). Enzyme-linked immune sorbent assay (ELISA) kits for HbA1c, GLP-1, and insulin were purchased from the Beijing Chenglin Bioengineering Institute (Beijing, China).

### Bacterial Strains and Growth Conditions

All *Lactobacillus* spp. strains used in this study were isolated from traditional fermented products and stored in the Key Laboratory of Dairy Science (KLDS) of the Northeast Agricultural University (NEAU), Ministry of Education, China. *L. rhamnosus* GG (ATCC 53103; Valio Ltd., Helsinki, Finland) served as the reference strain. All strains were anaerobically incubated in de Man Rogosa and Sharpe (MRS) broth (2% v/v) at 37°C for 18 h and subcultured twice prior to use.

### Preparation of Cell-Free Supernatants, Extracts, and Excretory Supernatants

After incubation, the cell-free supernatant (CFS) was harvested by centrifugation at 8,000 × *g* for 15 min at 4°C. The intact cells were washed three times with phosphate-buffered saline (PBS) solution (pH 7.4), after which the cells were resuspended in PBS and adjusted to 1.0 × 10^9^ CFU/ml. After that, cell-free extracts (CFE) were obtained by ultrasonic, which worked in 3–5-s pulses for 15 min in an ice bath. The cell fractions were removed by centrifugation at 8,000 × *g* for 15 min. The CFS and CFE were filter-sterilized with 0.22-μm filter membranes and stored in −80°C for further assays.

Cells of the strains were harvested by centrifugation (15 min, 8,000 × *g*, 4°C) after incubation at 37°C for 18 h. The cell pellets were rinsed three times with PBS and adjusted to 1 × 10^9^ CFU/ml and further incubated for 12 h. Cell-free excretory supernatants (CFES) were obtained by centrifugation at 8,000 × *g* for 15 min and kept in −80°C for further assay.

### Determination of DPP-IV Inhibition

The effect of the *Lactobacillus* on DPP-IV activity was determined by following the method of Lacroix and Li-Chan (2013) with some modifications. Briefly, in a 96-well microplate, 25 μl gly-pro-*p*-nitroanilide (0.2 mM, Sigma–Aldrich, St. Louis, MO, United States) and 25 μl bacterial sample (CFES, CFE) or 25 μl PBS as a control or diprotin A as a reference inhibitor were preincubated at 37°C for 10 min. Afterward, 50 μl DPP-IV (0.01 U/ml) was added and incubated at 37°C for 60 min. The reactions were terminated by addition of 100 μl sodium acetate buffer (1 M, pH 4.0), and the absorbance of the samples was measured at 405 nm by Infinite^@^ M1000 PRO ELISA plate reader (Tecan, Switzerland). Each sample was measured in triplicate, and the absorbance values were normalized to sample blanks in which DPP-IV was replaced with Tris–HCl buffer (100 mM, pH 8.0). The negative control (no DPP-IV activity) and positive control (DPP-IV activity with no inhibitor) were prepared by using Tris–HCl buffer (100 mM, pH 8.0) in place of the sample and DPP-IV solution, respectively. The DPP-IV inhibition rate (DIR) was calculated as follows:

$$\text{DIR}\left(\%\right)=\left(1-\frac{A_{\text{sample}}-A_{\mathrm{sample}\,\mathrm{blank}}}{A_{\mathrm{positive}\,\mathrm{control}}-A_{\mathrm{negative}\,\mathrm{control}}}\right)\times 100$$

### Determination of Antioxidative Activity of *Lactobacillus* Strains

#### Reducing Activity of Strains

Reducing activity was assessed as previously described (Oyaizu, 2010). First, 0.5 ml of samples was mixed with 0.5 ml of potassium ferricyanide (1%) and PBS (pH 6.6). The mixture was incubated at 50°C for 20 min and cooled rapidly, after which 0.5 ml of 10% trichloroacetic acid (TCA) was added. Next, the solution was centrifuged at 3,000 × *g* for 5 min. The supernatant (1.0 ml) was then mixed with 1.0 ml of 0.1% ferric chloride. The absorbance was measured at 700 nm after the mixture stood for 10 min. L-Cysteine hydrochloride was used as the standard expression for the reducing activity.

### 1,1-Diphenyl-2-Picryl-Hydrazyl Free Radical-Scavenging Activity

The 1,1-diphenyl-2-picryl-hydrazyl (DPPH) free radical-scavenging capacity of strains was determined using a modified method as previously described (Lin and Yen, 1999). Briefly, 1.0 ml of the CFS, CFE, or PBS (control group) was mixed with 1.0 ml of ethanolic DPPH radical solution (0.2 mM) or ethanol (blank group). The reaction solution was mixed and incubated at room temperature in the dark for 30 min. The scavenged DPPH was determined by measuring the decrease in absorbance at 517 nm after centrifugation at 6,000 × *g* for 10 min. The scavenging ability was calculated as follows:

$$\mathrm{Scavenging}\mathrm{activity}\left(\%\right)=\left(1-\frac{A_{\text{sample}}-A_{\text{blank}}}{A_{\text{control}}}\right)\times 100$$

### Hydroxyl Radical Scavenging Activity

The hydroxyl radical scavenging ability was analyzed by an assay as earlier described (Gutteridge, 1987). The reaction mixture contained 1.0 ml of 1,10-phenanthroline solution (2.5 mM), 1.0 ml of PBS (pH 7.4), 1.0 ml of samples, and 1.0 ml of FeSO_4_ (2.5 mM). Then, the reaction was initiated by the addition of 1.0 ml of 20 mM H_2_O_2_ and incubated at 37°C for 90 min. The absorbance of the solution was measured at 517 nm. The scavenging ability of hydroxyl radical was expressed as follows:

$$\mathrm{Scavenging}\mathrm{effect}\left(\%\right)=\left(A_{\text{sample}}-A_{\text{blank}}\right)/\left(A_{\text{control}}-A_{\text{blank}}\right)\times 100$$

### Superoxide Anion Radical Scavenging Activity

The superoxide anion radical scavenging ability was evaluated following the method of Li et al. (2012), with some modifications. First, 1.0 ml of the sample or deionized water (control group) was added to 3.0 ml of Tris–HCl solution (pH 8.2). The reaction mixture was incubated at 25°C for 20 min. In the following, 0.4 ml of pyrogallol (25 mM) was added, and the mixture was maintained at room temperature for 4 min. Then, reactions were terminated by adding 0.5 ml of HCl, and the absorbance was measured at 325 nm. The superoxide anion radical scavenging activity was defined as:

$$\mathrm{Scavenging}\mathrm{activity}\left(\%\right)=\left(1-\frac{A_{\text{sample}}}{A_{\text{blank}}}\right)\times 100$$

### Lipid Peroxidation Inhibiting Capacity

The lipid peroxidation inhibition capacity was conducted as earlier described by Lin and Chang (2000), with slight modifications. Briefly, 0.5 ml of PBS (pH 7.4), 1.0 ml of linoleic acid emulsion, 0.2 ml of FeSO_4_ (0.01%), 0.2 ml of ascorbic acid (0.01%), and 0.5 ml of samples were mixed and incubated at 37°C for 12 h. Then, 2.0 ml of 0.8% TBA, 0.2 ml of 4% TCA, and 0.2 ml of butylated hydroxytoluene (BHT) were added, and the mixture was incubated at 100°C for 30 min in a water bath. After cooling, 2.0 ml of chloroform was added, and the upper extract was obtained by centrifugation at 6,000 × *g* for 10 min. The inhibition of linoleic acid peroxidation was expressed as follows:

$$\mathrm{Inhibiting}\mathrm{effect}\left(\%\right)=\left(\frac{A_{\text{control}}-A_{\text{sample}}}{A_{\text{control}}}\right)\times 100$$

### Acid Resistance of Strains

Resistance to acidic conditions was assessed by a modified method as previously reported (Buntin et al., 2008). Cells that were cultured in MRS broth overnight at 37°C were collected by centrifugation (8,000 × *g*) for 15 min and then resuspended in PBS (pH 3.0 or 2.0). The tubes were incubated at 37°C, and the viable cells were counted by serial dilution and the pour plate method on MRS agar at 0, 1, 2, and 3 h.

### Bile Tolerance of Strains

Bile tolerance of the strains was determined using a modified method as recently described (Guo et al., 2016). *Lactobacillus* strains were grown at 37°C for 18 h in MRS broth without bile and then cells were resuspended in MRS broth with or without bile salt concentrations of 0.3% (w/v) at 37°C. Absorbance at 620 nm was measured every hour. The bile tolerance of each strain was based on the time required to increase the absorbance at 620 nm by 0.3 units. The delay of growth in time between the culture media was defined as the lag time (LT).

### Hydrophobicity of Strains

This was evaluated using *p*-xylene and ethyl acetate by a modified method as previously reported (Rosenberg et al., 2010). Briefly, the cell suspension was adjusted to an absorbance value (*A* 610) of approximately 0.8–1.0. The bacterial suspension (3.0 ml) was mixed with 1.0 ml of hydrocarbons, and the mixture was preincubated at 37°C for 10 min and then vortexed for 120 s. The suspension was then maintained at 37°C for 1 h. The aqueous phase was removed carefully, and the absorbance was measured using spectrophotometer (DU 800, Beckman Coulter, United States). Cell surface hydrophobicity (% CSH) was expressed as:

$$\%\text{CSH}=\frac{{\text{OD}}_{\text{Initial}}-{\text{OD}}_{\text{Final}}}{{\text{OD}}_{\text{Final}}}\times 100$$

where OD initial and OD final are the absorbance before and after extraction with the two hydrocarbons.

### Animals Studies

Male C57BL/6J mice (3 weeks old) were purchased from the Vital River Laboratory Animal Technology Company (Beijing, China). The model of mice with T2D was induced by HFD and intraperitoneal injection of STZ (Srinivasan and Ramarao, 2007; Chen et al., 2014a). All mice were housed in a special room with controlled temperature (22 ± 2°C), humidity (55 ± 5%), and light (7:00–19:00). All animals were fed *ad libitum* for 1 week, and then 15 mice were fed normal chow diet (NCD) and 45 mice were fed HFD (Supplementary Table S2). The dietary treatments continued for 5 weeks of the study. After 4 weeks of dietary intervention, all mice received intraperitoneal injection after fasting for 12 h. The NCD-fed mice received intraperitoneal injection with 50 mmol/L citrate buffer (pH 4.5), and the HFD-fed mice received STZ (Sigma, St. Louis, MO, United States), which dissolved in 50 mmol/L citrate buffer at a dose of 100 mg/kg of body weight (BW). A week later, tail blood glucose level was determined by glucometer (Roche Diagnostics, Germany). Mice with FBG level ≥ 11.1 mmol/L were defined as T2D.

### Experimental Design

Type 2 diabetes mice were randomly divided into three groups (*n* = 8 each): diabetes control group (DC), *L. acidophilus* KLDS1.0901-treated group (LA, 1 × 10^9^ CFU/day), and *L. rhamnosus* GG-treated group (LG, 1 × 10^9^ CFU/day). Eight mice fed with NCD served as the normal control group (NC). The DC and NC groups were treated with sterile PBS. All treatments were conducted with 10 ml per kilogram BW by oral gavage once daily for 6 weeks (Tian et al., 2016; Tonucci et al., 2017a). During weeks 6–11, all mice were fed a normal diet. BW, food consumption, and FBG were monitored weekly after fasting overnight. The NEAU Institutional Animal Care and Use Committee approved this study (Approval No.: SRM-06).

### Collection and Processing of Samples

After 12 weeks of treatment, all mice were sacrificed with ether anesthesia. The serum samples were obtained by centrifugation at 4,000 × *g* for 10 min and stored at *−*80°C for further analyses.

### Oral Glucose Tolerance Test

After a 12-h fasting period, an oral glucose tolerance test (OGTT) was performed on the last day of weeks 5 and 11. Glucose (2 g/kg BW) was orally administered to the mice. Blood samples were collected from the tail at 0, 30, 60, 90, and 120 min after glucose load, and glucose levels were measured. The area under the curve (AUC) was calculated by the linear trapezoid method (Reed et al., 2000).

### Biochemical Parameters

The levels of serum insulin, HbA1c, and GLP-1 were determined using ELISA kits (Beijing Chenglin Bioengineering Institute, Beijing, China) according to the manufacturer’s instructions. The activities of SOD and GSH-Px and level of GSH and MDA in the serum were measured with the assay kits (Nanjing Jiancheng Bioengineering Institute, Nanjing, China) following the manufacturer’s instructions.

### Statistical Analysis

Data are expressed as the mean ± standard deviation (*n* = 3 independent experiments) *in vitro* and (*n* = 8 independent experiments) *in vivo*. Statistical significance of difference was determined using one-way analysis of variance (ANOVA, SPSS 17.0) followed by multiple comparisons with Duncan’s multiple range test. Values of *p* < 0.05 were considered to be statistically significant.

## Results

### DPP-IV Inhibition by *Lactobacillus*

The DPP-IV inhibitory activities of the strains are shown in Table 1. The DIRs of the CFES samples ranged from 0 to 7.13%, with *L. acidophilus* KLDS1.1003 showing significantly higher level of inhibition (*p* < 0.05), while *L. rhamnosus* GG exhibited no inhibition activity. The range of DIR of CFE was from 0 to 55.42%. The greatest inhibitory activity (*p* < 0.05) was found in *L. acidophilus* KLDS1.0901 and followed by *L. acidophilus* KLDS1.1003. However, the CFE of *L. rhamnosus* GG performed lower DPP-IV inhibitory potential.

**TABLE 1:** Dipeptidyl peptidase IV (DPP-IV) inhibition (%) by *Lactobacillus* strains.

**Strains**	**DPP-IV inhibition rate (%)**	
	**CFES**	**CFE**
*L. rhamnosus* GG	ND	6.2 ± 1.4^f,g^
*L. rhamnosus* KLDS1.0205	3.0 ± 0.2^c^	10.0 ± 1.4^d,e,f^
*L. rhamnosus* KLDS1.0911	ND	11.1 ± 1.4^d,e^
*L. rhamnosus* KLDS1.0912	1.9 ± 0.2^d,e^	8.8 ± 1.8^e,f^
*L. plantarum* KLDS1.0317	1.9 ± 0.1^d,e^	9.5 ± 2.0^d,e,f^
*L. plantarum* KLDS1.0318	2.2 ± 0.2^d^	6.9 ± 0.6^e,f,g^
*L. plantarum* KLDS1.0344	ND	7.3 ± 0.9^e,f,g^
*L. plantarum* KLDS1.0386	0.4 ± 0.1^g^	3.6 ± 0.3^g^
*L. acidophilus* KLDS1.1003	7.1 ± 0.3^a^	50.1 ± 3.3^b^
*L. acidophilus* KLDS1.0901	1.7 ± 0.3^e^	55.4 ± 3.6^a^
*L. acidophilus* KLDS1.0902	1.2 ± 0.1^f^	13.1 ± 1.0^d^
*L. paracasei* KLDS1.0351	2.6 ± 0.2^c^	8.8 ± 1.9^e,f^
*L. helveticus* KLDS1.0903	3.1 ± 0.3^c^	21.1 ± 2.7^c^
*L. bulgaricus* KLDS1.0207	5.5 ± 0.3^b^	ND

*CFE, cell-free extract; CFES, cell-free excretory supernatant; ND, not detected. Values are expressed as mean ± SD (*n* = 3 independent experiments). Significant differences (*p* < 0.05) among different strains are indicated with different superscript letters.*

### Assay of Antioxidative Activity of *Lactobacillus* Strains

#### Reducing Activity

Strains exhibited varying degrees of reducing activity (Table 2). The CFE of *L. acidophilus* KLDS1.0902 exhibited the reducing activity that was equivalent to159.69 mmol of cysteine, significantly higher than that of *L. rhamnosus* GG (71.92 mmol) (*p* < 0.05). The CFS of *L. acidophilus* KLDS1.1003 possessed the highest reducing activity by 189.69 mmol of cysteine, no significant difference with that of *L. rhamnosus* GG (185.81 mmol). *L. plantarum* KLDS1.0317 showed the lowest reducing activity (73.31 mmol).

**TABLE 2:** Antioxidative activity of *Lactobacillus* strains.

**Strains**	**Reducing activity [equivalent**		**Scavenging of**		**Scavenging of**		**Scavenging of**		**Lipid peroxidation**	
	**cysteine (mmol)]**		**DPPH (%)**		** OH (%)**		**O_2_ (%)**		**inhibition capacity (%)**	
					
	**CFS**	**CFE**	**CFS**	**CFE**	**CFS**	**CFE**	**CFS**	**CFE**	**CFS**	**CFE**

LGG	168.0 ± 2.6^c^	71.9 ± 1.2^i^	77.7 ± 5.3^a^	26.6 ± 0.2^e^	64.0 ± 2.9^b,c^	33.8 ± 1.4^e^	22.6 ± 0.4^a^	18.9 ± 0.1^k^	22.3 ± 1.2^a^	14.3 ± 0.4^f^
KLDS1.0205	169.7 ± 2.6^b,c^	107.2 ± 2.8^e^	61.5 ± 0.3^c^	24.9 ± 0.2^f^	64.7 ± 3.1^a,b,c^	15.9 ± 1.9^i^	9.7 ± 0.2^h,i^	34.6 ± 0.5^f^	4.3 ± 0.8^f^	20.4 ± 0.9^d^
KLDS1.0911	144.1 ± 2.83^e^	120.8 ± 2.7^c^	50.6 ± 0.3^d^	20.3 ± 0.4^k^	62.5 ± 2.2^c^	29.9 ± 1.0^f^	12.5 ± 0.1^f^	33.1 ± 0.1^g^	11.0 ± 1.0^c^	31.4 ± 2.1^b^
KLDS1.0912	175.2 ± 3.5^b^	101.6 ± 1.0^f^	49.1 ± 0.6^d^	21.8 ± 0.1^i^	54.5 ± 1.2^e^	38.8 ± 1.7^c^	16.8 ± 0.1^c^	19.2 ± 0.3^j^	ND	19.6 ± 0.2^d^
KLDS1.0317	73.3 ± 2.4^i^	53.3 ± 1.4^k^	36.8 ± 0.3^e^	12.9 ± 0.3^n^	60.6 ± 1.9^c,d^	25.2 ± 0.9^g,h^	6.2 ± 0.1^k^	39.9 ± 0.4^b^	3.8 ± 1.4^f^	6.5 ± 0.7^g^
KLDS1.0318	93.0 ± 6.5^h^	60.8 ± 1.6^j^	49.6 ± 0.1^d^	20.9 ± 0.3^j^	44.0 ± 3.0^g^	57.6 ± 0.2^a^	13.4 ± 0.2^e^	43.3 ± 0.1^a^	11.9 ± 0.4^c^	5.9 ± 0.6^g^
KLDS1.0344	165.0 ± 1.0^c^	135.3 ± 1.8^b^	61.3 ± 0.3^c^	28.6 ± 0.2^c^	45.8 ± 1.6^g^	27.8 ± 0.9^f,g^	17.3 ± 0.2^b^	14.0 ± 0.1^m^	11.3 ± 0.9^c^	25.8 ± 2.0^c^
KLDS1.0386	109.4 ± 2.4^g^	53.0 ± 1.4^k^	49.6 ± 0.3^d^	23.3 ± 0.1^g^	50.0 ± 1.1^f^	38.3 ± 2.6^c^	10.6 ± 0.5^g^	35.0 ± 0.1^e^	3.6 ± 0.3^f^	7.7 ± 0.4^g^
KLDS1.1003	189.7 ± 2.7^a^	92.7 ± 1.2^g^	61.3 ± 0.1^c^	27.8 ± 0.1^d^	68.6 ± 0.7^a^	24.1 ± 1.3^h^	6.1 ± 0.3^k^	35.8 ± 0.3^d^	18.5 ± 0.6^b^	19.0 ± 1.4^d,e^
KLDS1.0901	152.7 ± 3.0^d^	88.9 ± 0.8^h^	72.7 ± 0.3^b^	32.8 ± 0.2^b^	67.7 ± 1.6^a,b^	53.8 ± 0.5^b^	9.3 ± 0.2^i^	31.4 ± 0.2^i^	8.8 ± 1.0^d^	16.7 ± 1.3^e^
KLDS1.0902	168.9 ± 2.4^b,c^	159.7 ± 1.0^a^	51.3 ± 0.1^d^	22.8 ± 0.3^h^	33.0 ± 1.6^h^	ND	2.6 ± 0.1^l^	18.1 ± 0.1^l^	1.5 ± 0.3^g^	25.3 ± 1.3^c^
KLDS1.0351	158.3 ± 2.4^d^	116.6 ± 0.4^d^	38.9 ± 0.2^e^	13.6 ± 0.2^m^	13.7 ± 0.9^i^	27.4 ± 1.7*f*^g,h^	10.0 ± 0.1^h^	38.7 ± 0.4^c^	8.5 ± 0.4^d,e^	12.3 ± 0.6^f^
KLDS1.0903	130.0 ± 2.0^f^	54.1 ± 0.4^k^	49.4 ± 0.2^d^	16.6 ± 0.2^l^	57.0 ± 2.2^d,e^	37.4 ± 2.9^c,d^	7.9 ± 0.3^j^	31.9 ± 0.1^h^	7.1 ± 0.4^e^	12.0 ± 0.1^f^
KLDS1.0207	185.8 ± 2.8^a^	119.1 ± 0.8^c,d^	62.1 ± 0.5^c^	34.5 ± 0.3^a^	57.8 ± 1.3^d,e^	34.7 ± 2.0^d,e^	16.2 ± 0.1^d^	12.2 ± 0.3^n^	5.1 ± 0.2^f^	51.0 ± 1.9^a^

*CFS, cell-free supernatant; CFE, cell-free extract; ND, not detected. Values expressed as mean ± SD (*n* = 3 independent experiments). Statistical significance was determined between different *Lactobacillus* strains on antioxidative activity. Data with different superscript letters in each row are significant (*p* < 0.05).*

### DPPH Free Radical-Scavenging Ability

In this study, all tested strains exhibited distinct DPPH radical-scavenging activity (Table 2). The CFE of *L. bulgaricus* KLDS1.0207 had the highest DPPH radical-scavenging activity with 34.46%, followed by *L. acidophilus* KLDS1.0901 (32.85%); *L. plantarum* KLDS1.0317 exhibited the lowest DPPH radical-scavenging activity (12.93%). Moreover, the CFS of strains exhibited higher DPPH radical-scavenging activity than that of CFE.

### Hydroxyl Radical Scavenging Ability

The CFS and CFE of all strains possessed the ability to eliminate the hydroxyl radical (Table 2). Among these strains, the CFE of *L. plantarum* KLDS1.0318, KLDS1.0386, *L. acidophilus* KLDS1.0901, *L. rhamnosus* KLDS1.0912, and *L. helveticus* KLDS1.0903 had better abilities to eliminate the hydroxyl radical, showing higher values than that of *L. rhamnosus* GG (*p* < 0.05). The results also showed that the CFS of *L. acidophilus* KLDS1.0901 and KLDS1.1003 had higher scavenging ability on the hydroxyl radical than that of *L. rhamnosus* GG.

### Superoxide Anion Radical-Scavenging Ability

In this study, strains showed different O_2_-scavenging activity ranging from 12.17 to 43.28% of CFE and 2.56 to 22.61% of CFS (Table 2). Among these strains, ${\text{O}}_{2}^{-}$-scavenging activity of CFE of test strains was higher than that of *L. rhamnosus* GG, except three strains (*L. acidophilus* KLDS1.0902, *L. plantarum* KLDS1.0344, and *L. bulgaricus* KLDS1.0207).

### Lipid Peroxidation Inhibition Capacity

There were significant differences in the lipid peroxidation inhibition capacities of the screened strains (Table 2). The CFE of *L. bulgaricus* KLDS1.0207 had highest inhibition capacity of 51.04%. On the other hand, *L. plantarum* strains KLDS1.0317, KLDS1.0318, and KLDS1.0386 exhibited lower inhibition rates (<10%). In addition, the CFS of all *Lactobacillus* strains showed lower inhibition capacity than that of the reference strain *L. rhamnosus* GG.

### Principal Component Analysis

**FIGURE 1:**
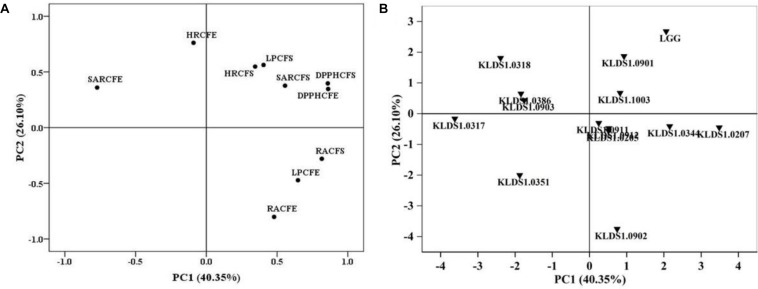
Loading and score plots of principal component analysis. **(A)** Loading plot for results of five different antioxidant assays. RACFS (CFE), reducing activity of cell-free supernatant (CFS) [cell-free extract (CFE)]; DPPH CFS (CFE), 1,1-diphenyl-2-picryl-hydrazyl (DPPH)-scavenging activity of CFS (CFE); HRCFS (CFE), hydroxyl radical-scavenging activity of CFS (CFE); SARCFS (CFE), superoxide anion radical-scavenging activity of CFS (CFE); LPCFS (CFE), lipid peroxidation inhibition capacity of CFS (CFE). **(B)** Score plot of the antioxidative properties of *Lactobacillus* strains. PC1, first principal component; PC2, second principal component.

Principal component analysis (PCA) was used to evaluate the antioxidative activities of all strains in this study. Two independent principal components (PCs) were extracted. As shown in Figure 1A, PC1, which explained 40.35% of the total variance, was characterized by reducing activity of CFS, DPPH free radical scavenging of CFE, and superoxide anion radical scavenging of CFE. PC2, which accounted for 26.10% of the total variance, was mainly related to reducing activity of CFS and hydroxyl radical-scavenging ability of CFE. The strain score plot for PC1 versus PC2 is presented in Figure 1B. In addition, the total score was used to classify all tested strains (Supplementary Table S1). The total scores of *L. rhamnosus* GG, *L. plantarum* KLDS1.0344, *L. acidophilus* KLDS1.1003, *L. acidophilus* KLDS1.0901, and *L. bulgaricus* KLDS1.0207 were 1.91, 0.85, 0.92, 1.23, and 1.60, respectively, which were higher than those of other strains. Based on these results, these five strains were selected for further study.

### Acid and Bile Salt Tolerance of Strains

**FIGURE 2:**
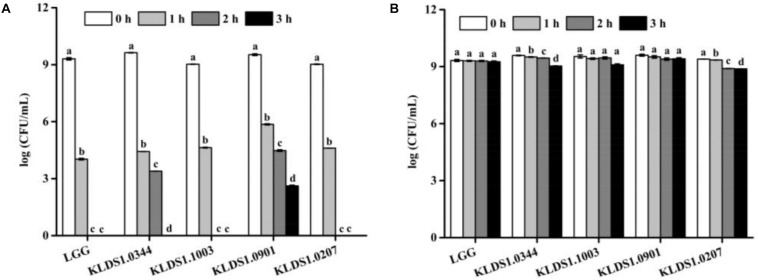
Effect of pH 2.0 and pH 3.0 on viability of *Lactobacillus* strains. Graph **(A)** presents the effect of pH 2.0 on viability of *Lactobacillus* strains whereas Graph **(B)** presents the effect of pH 3.0 on viability of *Lactobacillus* strains. Values represent mean ± SD (*n* = 3 independent experiments). Significant differences (*p* < 0.05) among different groups are indicated with different superscript letters.

Acid tolerance patterns of the *Lactobacillus* strains were assessed *in vitro* (Figure 2). The survival rates of the five *Lactobacillus* strains were close to 100% at pH 3.0. In acidic environment with at pH 2.0, the survival rates of the five *Lactobacillus* strains decreased significantly within 3 h; only *L. acidophilus* KLDS1.0901 could survive for 3 h under this environment. These results suggested that *L. acidophilus* KLDS1.0901 had better acid resistance than other strains. All studied strains were tolerant to 0.3% (w/v) of bile salt (Table 3). The LTs of *L. rhamnosus* GG, *L. plantarum* KLDS1.0344, *L. acidophilus* KLDS1.1003, *L. acidophilus* KLDS1.0901, and *L. bulgaricus* KLDS1.0207 were 2.06, 2.46, 2.26, 2.44, and 3.27 h, respectively. The results suggested that *L. rhamnosus* GG had better bile salt resistance than other strains. In addition, there was no significant difference between *L. acidophilus* KLDS1.0901 and *L. acidophilus* KLDS1.1003 (*p* > 0.05).

**TABLE 3:** Comparison of *Lactobacillus* for their bile salt and acid tolerance and hydrophobicity.

**Strains**	**Bile salt tolerance (T/h)**	**Hydrophobicity**	
		**Xylene**	**Ethyl acetate**
LGG	2.0 ± 0.16^c^	4.71 ± 0.37^b^	17.75 ± 1.07^c^
KLDS1.0344	2.46 ± 0.12^b^	ND	42.42 ± 2.03^a^
KLDS1.1003	2.26 ± 0.13^b,c^	134.33 ± 1.13^a^	20.51 ± 0.54^c^
KLDS1.0901	2.44 ± 0.10^b^	130.58 ± 2.82^a^	30.84 ± 2.07^b^
KLDS1.0207	3.27 ± 0.10^a^	2.51 ± 0.04^b^	18.30 ± 0.89^c^

*T (h)-required for absorbance at 620 nm to increase by 0.3 units in each medium. Each value in the table is the mean ± SD (*n* = 3 independent experiments). Means with different superscript letters in the same column per treatment indicate significant difference (*p* < 0.05).*

### Hydrophobicity of Strains

The hydrophobicity levels of the five selected strains are also reported (Table 3). Strains’ CSH values were higher in xylene solution than in ethyl acetate solution. In xylene solution, the CSH of strains ranged from 0 to 134.33%. It is interesting to note that *L. acidophilus* KLDS1.1003 (134.33%) and *L. acidophilus* KLDS1.0901 (130.58%) had higher hydrophobicity than the reference strain *L. rhamnosus* GG (4.71%). In ethyl acetate solution, the CSH of strains ranged from 17.75 to 42.42%, and the hydrophobicity levels of two test strains, *L. acidophilus* KLDS1.0901 (30.84%) and *L. plantarum* KLDS1.0344 (42.42%), were higher than that of *L. rhamnosus* GG (17.75%). These results suggested that *L. acidophilus* KLDS1.0901 had a better average hydrophobicity level than other strains.

### *In vivo* Effect of *Lactobacillus* on Diabetic Mice

#### Effect of *Lactobacillus* on Body Weight and Food Consumption

**FIGURE 3:**
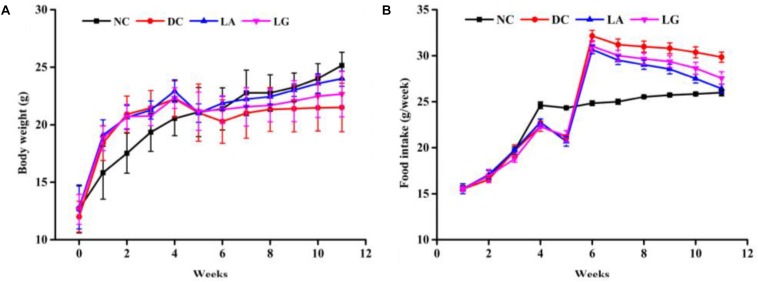
Effect of probiotics feeding on **(A)** body weight and **(B)** food intake. NC, normal control group; DC, diabetic control group; LG, DC plus *L. rhamnosus* GG; LA, DC plus *L. acidophilus* KLDS1.0901.

Based on the *in vitro* results, *L. acidophilus* KLDS1.0901 was selected to further evaluate its antidiabetic effects *in vivo*. Changes in BW and food intake of study animals were recorded for 12 weeks (Figure 3). After STZ injection, weight loss was observed in all HFD groups. However, the T2D mice that received *L. acidophilus* KLDS1.0901 or *L. rhamnosus* GG showed a significant (*p* < 0.05) increase in BW compared to the DC group. This trend was observed until the end of the experiment (Figure 3A). The T2D mice had a significant (*p* < 0.05) increase in food intake compared with the normal mice from weeks 1 to 4. Administering STZ injection induced higher food intake in HFD mice, whereas food intake of the LA group was significantly lower compared with the DC group from week 6 to week 11.

#### Effect of *Lactobacillus* on Glucose Tolerance and FBG

**FIGURE 4:**
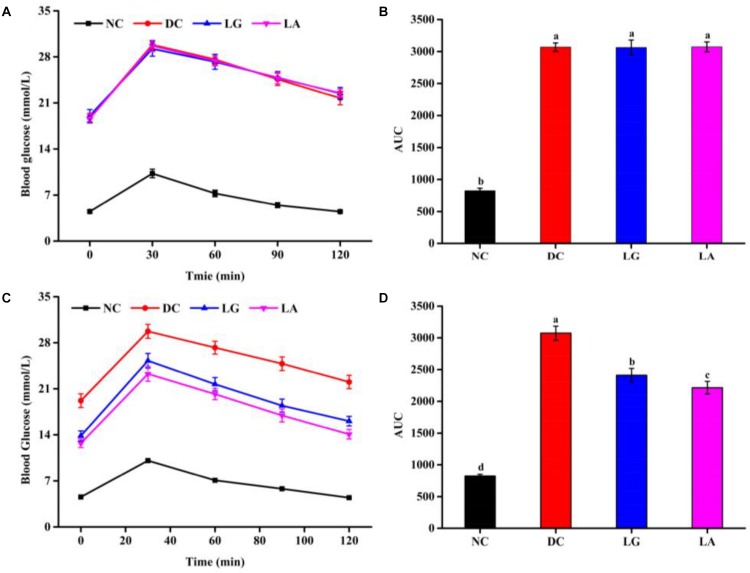
The effect of probiotic treatments on oral glucose tolerance (OGTT) and AUC_glucose_ at weeks 5 and 11. NC, normal control group; DC, diabetic control group; LG, DC plus *L. rhamnosus* GG; LA, DC plus *L. acidophilus* KLDS1.0901. **(A)** OGTT at week 5. **(B)** AUC_glucose_ at week 5. **(C)** OGTT at week 11. **(D)** AUC_glucose_ at week 11. All data are expressed as the mean ± SD (*n* = 8 independent experiments). Groups without common letters differ significantly from one another (*p* < 0.05).

At week 5, the blood glucose level at different times and AUC_glucose_ of the DC groups were higher than those in the NC group (Figures 4A,B). These results indicated that glucose tolerance was impaired in the DC group, and the diabetic models were established. At week 11, the glucose tolerance was clearly improved by *L. acidophilus* KLDS1.0901 and *L. rhamnosus* GG administration (Figures 4C,D). The glucose tolerance of the NC group remained stable. Furthermore, the blood glucose and AUC_glucose_ levels of the LA group were lower than that in the LG group, indicating that the glucose tolerance of the LA group was better than that of the LG group.

**FIGURE 5:**
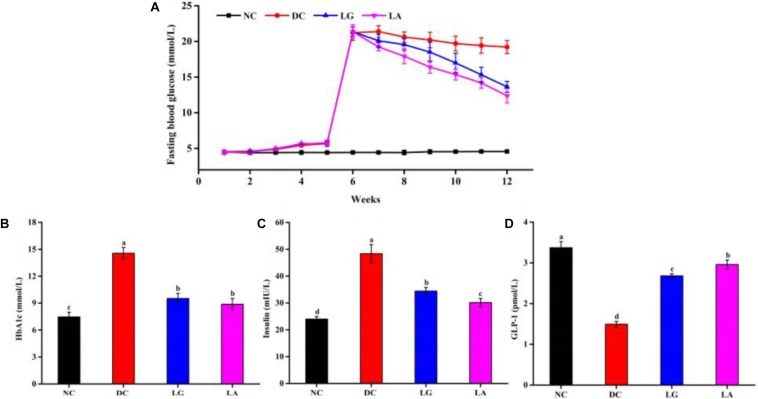
Effects of probiotics on fasting blood glucose **(A)**, serum HbA1c **(B)**, insulin **(C)**, and GLP-1 **(D)** levels. NC, normal control group; DC, diabetic control group; LG, DC plus *L. rhamnosus* GG; LA, DC plus *L. acidophilus* KLDS1.0901. Data are expressed as the mean ± SD (*n* = 8 independent experiments). Values with different letters are significantly different (*p* < 0.05).

Fasting blood glucose level of the HFD groups was increased after STZ injection (Figure 5A). Administration of *L. acidophilus* KLDS1.0901 or *L. rhamnosus* GG decreased FBG level from week 6 to week 11. At the end of week 11, the levels of FBG in the LA and LG groups were significantly lower than that in the DC group (*p* < 0.05), but still higher than that in the NC group.

#### Effect of *Lactobacillus* on HbA1c, Insulin, and GLP-1 of Blood Levels

At the end of the treatment, the levels of HbA1c and insulin in the DC group were significantly higher than that of the NC group (*p* < 0.05), whereas oral administration of *L. acidophilus* KLDS1.0901 or *L. rhamnosus* GG significantly reduced the HbA1c level in the LA and LG groups (*p* < 0.05) (Figures 5B,C). Moreover, the level of GLP-1 was significantly reduced in the DC group (*p* < 0.05) than that in the NC, LA, and LG groups. In addition, the level of GLP-1 in the LA group was higher than that in the LG group (*p* < 0.05) (Figure 5D). These again suggest that *L. acidophilus* KLDS1.0901 was more effective in attenuating HbA1c, insulin, and GLP-1 levels.

#### Effect of *Lactobacillus* on Oxidative Stress Status of Mice Serum

**FIGURE 6:**
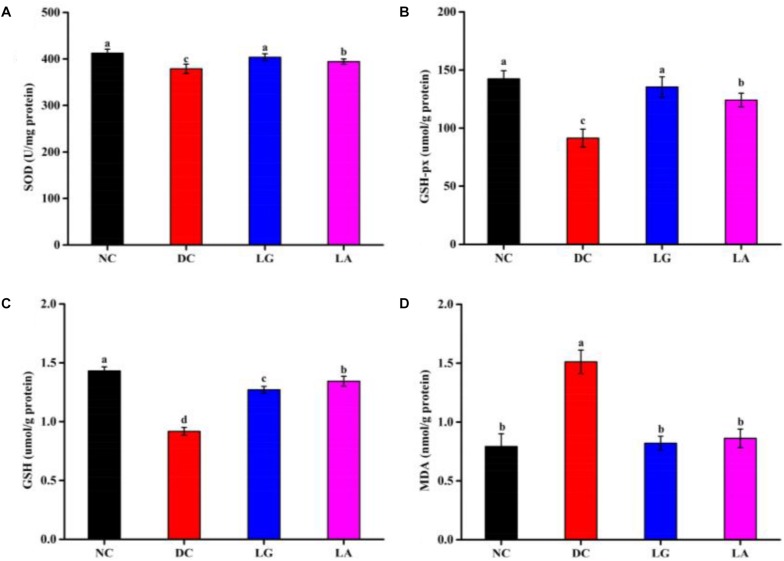
Effect of probiotic feeding on oxidative stress in blood. **(A)** Superoxide dismutase (SOD). **(B)** Glutathione peroxidase (GSH-Px). **(C)** Glutathione (GSH). **(D)** Malondialdehyde (MDA). NC, normal control group; DC, diabetic control group; LG, DC plus *L. rhamnosus* GG; LA, DC plus *L. acidophilus* KLDS1.0901. Values are expressed as mean ± SD (*n* = 8 independent experiments). Values with different superscript letters are significantly different (*p* < 0.05).

Oxidative stress parameters (SOD, GSH-Px, GSH, and MDA) in serum are presented in Figure 6. The activities of SOD (378.93 U/mg protein) and GSH-Px (91.39 μmol/g protein) and the level of GSH (0.92 μmol/g protein) were significantly reduced (*p* < 0.05), and the level of MDA (1.51 nmol/g protein) was significantly increased in the DC group compared with the NC group (*p* < 0.05) (Figure 6). Also, the activities of SOD and GSH-Px were increased in the LA and LG groups compared with the DC group (*p* < 0.05). However, the level of GSH was higher in the LA group than that in the LG group. Moreover, lower MDA contents in the LA and LG groups were observed compared to the DC group, whereas no significant differences were observed between the LA and LG groups (*p* > 0.05).

## Discussion

A growing body of evidence suggests that consumption of some strains of *Lactobacillus* (*L. paracasei*, *L. plantarum*, *L. acidophilus*, *L. rhamnosus*, and *L. acidophilus*) or its fermentation products could alleviate diabetes (Tabuchi et al., 2003; Yadav et al., 2007; Li et al., 2014; Dang et al., 2018). Additionally, among these *Lactobacillus* spp. strains, several strains were obtained from traditional fermented products. In the present study, antidiabetic strains were selected from 14 *Lactobacillus* spp. strains, which were obtained from traditional fermented dairy or vegetable products. Previous study showed that the method of screening for hypoglycemic probiotics based on antioxidative and α-glucosidase inhibitory activity was reliable (Chen et al., 2014c). It is generally known that α-glucosidase, distributed on the brush border membrane of the small intestine, catalyzes the digestive process of carbohydrates (Hansawasdi et al., 2001). However, probiotics play a major role in the large intestine.

The DPP-IV enzyme is expressed in a variety of cells, particularly on epithelial tissues (Lambeir et al., 2003; Nongonierma and FitzGerald, 2016). It could inactivate GLP-1, which is important for glucose metabolism regulation. Additionally, DPP-IV inhibitors are the newest and most promising antidiabetic drugs and have fewer side effects compared with other agents (Reimer et al., 2002; Akoumianakis et al., 2018; Musoev et al., 2019). In recent years, DPP-IV inhibitors from natural sources have gradually become a safe and potential treatment for patients with hyperglycemia (Li N. et al., 2018; Parmar et al., 2012). Previous studies have reported that *Bacillus* and *Streptomyces* spp. showed DPPIV inhibitory activity. Diprotin A (DPP-4 inhibitor) was isolated from culture filtrates of *Bacillus cereus* BMF673-RF1 (Umezawa, 1984). Sulfostin S, a novel DPP-IV inhibitor, was isolated from the culture broth of *Streptomyces* sp. MK251-43F3 (Abe et al., 2005). Additionally, it has been demonstrated that hydrolysates and peptides from cow’s milk, bovine meat, and salmon (Lacroix and Li-Chan, 2012) were able to inhibit the activity of DPP-IV *in vitro*. In the present study, DPP-IV inhibitory activity was used as an indicator to screen antidiabetic strains. Most *Lactobacillus* spp. strains showed higher DPP-IV inhibitory activity with *L. acidophilus* KLDS1.1003 and *L. acidophilus* KLDS1.0901, which was consistent with other studies (Panwar et al., 2015; Zhu et al., 2016). However, it was observed that DPP-IV inhibitory levels were higher in previous studies; this may be due to different concentrations of bacteria or sources of DPP-IV. Findings from a previous study showed that porcine DPP-IV showed higher inhibition levels than the human DPP-IV (Lacroix and Li-Chan, 2015). In addition, the DPP-IV inhibitory activity of the CFS was not affected by temperature and pH; however, it was sensitive to proteases. Some casein or whey protein-derived peptides, such as dipeptides, tripeptides, and tetrapeptides, possessed DPP-IV inhibitory activity, suggesting that peptides might be responsible for DPP-IV inhibitory activity and that residual peptide levels in the CFES or CFE may have enzyme inhibitory activity (Lacroix and Li-Chan, 2013; Nongonierma and Fitzgerald, 2013). Furthermore, X-prolyl-dipeptidylamino-peptidase (PepX), a proline-specific peptidase, was almost identical to DPP-IV (Meyer-Barton et al., 1993). Interestingly, the PepX gene or PepX activity have been observed in *Lactobacillus* spp. strains, including *L. casei*, *L. delbrueckii*, *L. helveticus*, and *L. rhamnosus* (Habibi-Najafi and Lee, 1994; Savijoki et al., 2006), which may also provide a possible explanation for the DPP- IV inhibitory activity of bacteria.

Oxidative stress is thought to be a major characteristic in the development of diabetes (Rains and Jain, 2011). Previous studies have shown that lactic acid bacteria, especially *Lactobacillus* spp. strains, possess antioxidant activity (Tonucci et al., 2017a; Heshmati et al., 2018). The intact cells, CFE, and CFS, of *L. casei* CCFM0412 and *L. rhamnosus* CCFM0528 were found to scavenge hydroxyl radicals and DPPH-free radicals, inhibit linoleic acid peroxidation *in vitro*, and enhance the antioxidative activities of mice *in vivo* (Chen et al., 2014b, c). Studies have demonstrated that the antioxidant mechanisms of probiotic include chelating metal ion, possessing own antioxidant enzymatic systems, and producing metabolites with antioxidative activity, such as GSH, butyrate, and folate (Wang Y. et al., 2017). In this study, *L. bulgaricus* KLDS1.0207, *L. acidophilus* KLDS1.0901, and *L. acidophilus* KLDS1.1003 had higher antioxidative activity than other strains.

Surviving under gastrointestinal tract (GIT) conditions and colonizing the intestine are important for probiotics to offer health benefits to the host. In the present study, five strains of *Lactobacillus* have acid and bile salt tolerance (Table 3 and Figure 2). Moreover, adhesion to intestinal epithelial cells is an important prerequisite for colonizing probiotic strains in the GIT (Pedersen and Tannock, 1989; Carmen et al., 2009; Argyri et al., 2013). Previous studies have posited a direct correlation between hydrophobicity and adhesion to intestinal epithelial cells (Re et al., 2010). In this study, *L. acidophilus* KLDS1.0901 exhibited the best hydrophobicity, indicating that it may have the potential to colonize the intestine.

*Lactobacillus* with acid-tolerant, bile salt-tolerant, and good hydrophobic properties has potential for adhesion to or colonization of the host intestine and could continually play a role in improving immunity parameters. However, the therapeutic effect of CFE administration is short-lived and requires regular replenishing. Additionally, some studies have indicated that a part of LAB could be broken up in intestinal tract and release intracellular substances (Lin and Yen, 1999; Lin and Chang, 2000). Furthermore, many recent studies have reported that LAB could modulate gut microbiota, promoting the growth of the beneficial bacteria and inhibiting the growth of pathogens (Li et al., 2017; Li B. et al., 2018; Qu et al., 2018). In recent years, a large body of studies have also demonstrated that the occurrence and development of T2D were related to gut microbiota (Qin et al., 2012; Karlsson et al., 2013). Therefore, in the present study, living *L. acidophilus* KLDS1.0901 was selected as a candidate for further study in the T2D mice model. Previous studies have reported that oral administration of probiotics could reduce FBG and HbA1c levels, ameliorate oxidative stress, and improve insulin resistance of T2D mice (Manaer et al., 2015; Tonucci et al., 2017a). Therefore, in the present study, living strain was selected to feed mice. Results showed that *L. acidophilus* KLDS1.0901 treatment could significantly decrease food intake and FBG level and increase BW (Figure 3A), which is consistent with other studies (Li et al., 2016). HbA1c, which is a clinical diagnostic parameter for diabetes, reflects the average plasma glucose concentration over a period of time. *L. acidophilus* KLDS1.0901 significantly reduced the level of HbA1c, which indicated that *L. acidophilus* KLDS1.0901 could relieve long-time high blood glucose status. These results suggest that this strain could effectively improve T2D, and the methods of screening for antidiabetic probiotics *in vitro* were effective.

Insulin resistance is known to accelerate the occurrence and development of T2D, a typical characteristic of T2D (Liu et al., 2015). OGTT, which has often been used to estimate insulin resistance, is a well-established diagnostic criterion for T2D (Sharma and Srinivasan, 2009). In this study, after 6 weeks of treatment, the glucose intolerance was significantly improved in the LA and LG groups (Figure 4). Insulin is an important protein hormone that functions in the regulation of glucose metabolism and maintains a balance blood glucose level. Administration of *L. acidophilus* KLDS1.0901 significantly reduced the fasting insulin level than that of the DC group (Figure 5C). Moreover, it was reported that reduction in the levels of insulin and AUC_glucose_ is associated with improving insulin resistance. Thus, these results indicated that *L. acidophilus* KLDS1.0901 could ameliorate insulin resistance in T2D mice.

Glucagon-like peptide-1, an important incretin secreted by intestinal L-cells, plays an important role in approving T2D by lowering blood glucose and preserving pancreatic β-cell function (Puddu et al., 2014). DPP-IV, a proconvertase, inactivates the GLP-1 by removing the first two N-terminal amino acid residues (Nauck, 2011). Interestingly, *L. acidophilus* KLDS1.0901 and *L. rhamnosus* GG with DPP-IV inhibitory activity *in vitro* could increase the GLP-1 level in T2D mice. In addition, the level of GLP-1 in the LA group was significant higher than that in the LG group (Figure 5D). These results suggested that the screened strains were good DPP-IV inhibitors *in vitro* and could increase the level of GLP-1 *in vivo*.

It has been reported that oxidative stress plays an important role in the development of T2D. Hyperglycemia could increase oxidative stress; the imbalance of oxidative stress status could impair the living cell membrane and further promote the occurrence and development of diabetes (Hariom et al., 2007). Previous studies reported that abnormal SOD and GSH-Px activities as well as GSH and MDA level were found in STZ-induced diabetic rats or mice (Lee, 2006). MDA, a lipid peroxidation marker and an end-product of lipid peroxidation process, is toxic to DNA and protein (Li et al., 2014). The MDA level increases in the blood and organ under oxidative stress conditions (Niedowicz and Daleke, 2005). In our study, oral administration of *L. acidophilus* KLDS1.0901 in diabetic mice increased activities of SOD and GSH-Px and decreased the level of MDA (Figure 6D), which was in consonance with previous studies (Chen et al., 2014a). These results indicated that *L. acidophilus* KLDS1.0901 could ameliorate T2D by reducing oxidative stress status. Additionally, a recent study showed that *L. acidophilus* KLDS1.0901 could improve the epithelial barrier function, lower inflammation cytokines, and reshape the structure and composition of the gut microbiota, increasing the relative abundance of SCFA-producing bacteria and the level of SCFAs, especially butyric acid. Subsequently, butyric acid targets liver via the portal vein and activates the glucose and lipid metabolism-related signaling pathways (Yan et al., 2019).

## Conclusion

The need to explore potential anti-diabetic LAB strains is an important objective of many recent research interventions. This study showed that *L. acidophilus* KLDS1.0901 possesses high DPP-IV inhibitory activity and probiotic properties *in vitro*. Administration of *L. acidophilus* KLDS1.0901 could maintain the balance of blood glucose and ameliorate insulin resistance and oxidative stress in mice with T2D. Thus, this strain can serve as a novel probiotic in the manufacture of probiotic products, medications, and functional food that can lower FBG levels and attenuate T2D biomarkers.

## Data Availability Statement

All datasets generated for this study are included in the article/Supplementary Material.

## Ethics Statement

The animal study was reviewed and approved by the Institutional Animal Care and Use Committee of the Northeast Agricultural University (SRM-06).

## Author Contributions

GH and FY designed the study. BL supervised the whole experiments. FY, YY, and CW performed the experiments. FY, NL, and LZ analyzed the data. FY wrote the manuscript. SE revised the manuscript.

## Conflict of Interest

The authors declare that the research was conducted in the absence of any commercial or financial relationships that could be construed as a potential conflict of interest.
