# Supplementary material for: Screening for Potential Novel Probiotics With Dipeptidyl Peptidase IV-Inhibiting Activity for Type 2 Diabetes Attenuation in vitro and in vivo
Source: Front Microbiol. 2020 Jan 10;10:2855. doi: 10.3389/fmicb.2019.02855 (PMC6965065; doi:10.3389/fmicb.2019.02855)
Supplement: Supplementary file 1 [file Data_Sheet_1.pdf]

Supplementary Material

Table S1 The rating form of antioxidative ability of *Lactobacillus* spp. strains.

Strains	Score	Strains	Score
LGG	1.91	KLDS1.0386	-0.77
KLDS1.0205	0.26	KLDS1.1003	0.93
KLDS1.0911	0.05	KLDS1.0901	1.24
KLDS1.0912	-0.05	KLDS1.0902	-0.79
KLDS1.0317	-1.86	KLDS1.0351	-1.8
KLDS1.0318	-0.78	KLDS1.0903	-0.73
KLDS1.0344	0.85	KLDS1.0207	1.60

Table S2 Composition of normal chow and high fat diet

Diet	Normal diet	High fat diet
Energy composition(%)		
Carbohydrate	63.9%	35%
Protein	20.3%	20%
Fat	15.8%	45%
Ingredients (g/kg)		
Casein	200	200
L-Cystine	3	3
Corn starch	397	72.8
Maltodextrin	132	100
Sucrose	100	172.8
Cellulose	50	50
Soybean Oil	70	25
t-Butylhydroquinone	0.014	25
Lard Oil	0	177.5
Mineral Mix	35	10
Di Calcium Phosphate	0	13
Calcium Carbonate	0	5.5
Potassium Bitartrate	0	16.5
Vitamin Mix	10	10
Choline Bitartrate	2.5	2
FD&C Red Dye	0	0.05
